# Editorial: Interpersonal synchrony and network dynamics in social interaction

**DOI:** 10.3389/fnhum.2022.1095735

**Published:** 2022-11-29

**Authors:** Viktor Müller, Merle T. Fairhurst, Floris T. van Vugt, Peter E. Keller, Markus F. Müller

**Affiliations:** ^1^Center for Lifespan Psychology, Max Planck Institute for Human Development, Berlin, Germany; ^2^Faculty of Human Sciences, Institute for Psychology, Universität der Bundeswehr München, Neubiberg, Germany; ^3^Center for Tactile Internet with Human-in-the-Loop (CeTI), Technische Universität Dresden, Dresden, Germany; ^4^Department of Psychology, University of Montreal, Montreal, QC, Canada; ^5^Department of Clinical Medicine, Center for Music in the Brain, Aarhus University, Aarhus, Denmark; ^6^MARCS Institute for Brain, Behaviour and Development, Western Sydney University, Penrith, NSW, Australia; ^7^Centro de Investigación en Ciencias, Universidad Autónoma del Estado de Morelos, Cuernavaca, Morelos, Mexico

**Keywords:** interpersonal action coordination, hyperscanning, intra- and inter-brain synchronization, network topology and network dynamics, functional connectivity, group interaction

This Research Topic was launched with the aim of highlighting and exploring the mechanisms and functions of interpersonal interaction, and thereby deepening our understanding of these highly interesting and complex phenomena and their downstream effects on real-life social interaction. The collection of contributions includes a Hypothesis and Theory article, a Review, two Brief Research Reports, and 11 Original Research articles written by leading researchers in the field. They showcase the breadth of research studies, going from hyper-brain cell assembly hypothesis and theory of mind hyperscanning to ensemble singing and soccer playing to healthcare teams, music therapy and psychotherapy concepts based on the inter-brain plasticity model. This range exemplifies the promise of this field in being able to span multiple facets of life. Figure 1 illustrates the diversity yet thematic relatedness of the contributions. It displays the joint forward model for interpersonal action coordination with three representation levels (i.e., representation of individual, other's, and joint forward models with corresponding sensorimotor feedbacks) and the impact of a joint goal as well as external influences (cf. Müller et al., 2021). This model is a construct integrating and reflecting a variety of influences and interactions of human social behavior. Figure 1 also highlights different interaction situations described in the Research Topic.

**Figure 1:**
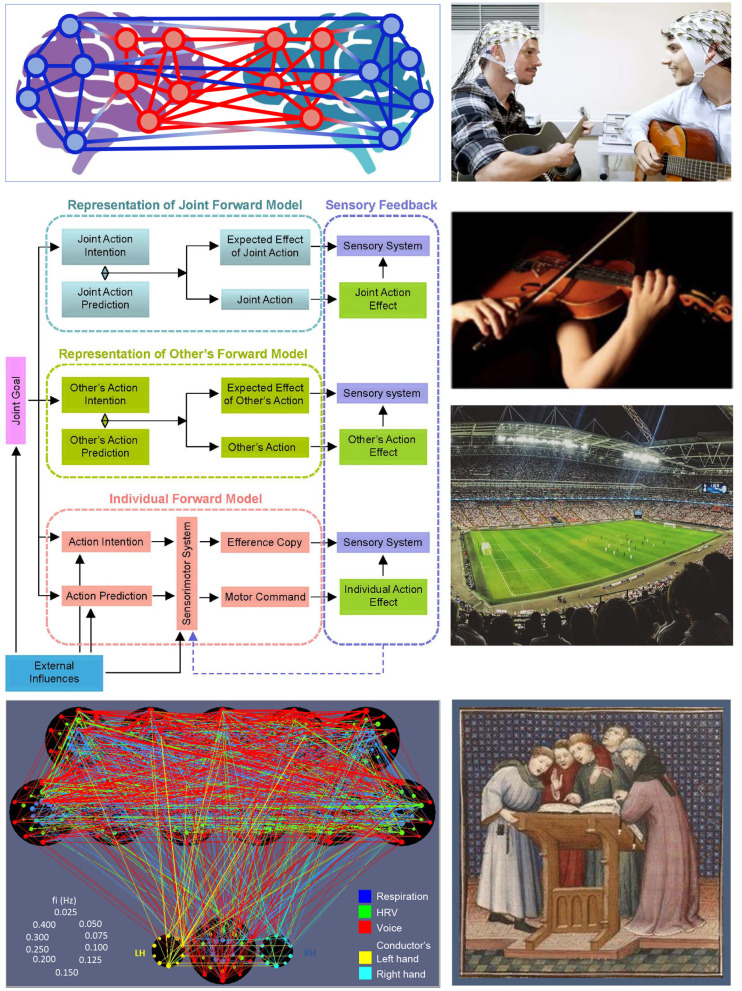
Joint forward model for interpersonal action coordination and various social interaction situations described in the Research Topic. The joint forward model for interpersonal action coordination with three representation levels (i.e., representation of individual, other's, and joint forward models with corresponding sensorimotor feedbacks) is represented in the middle left (adapted from Müller et al., 2021). This model reflects a variety of the interactions described in the Research Topic. On the bottom left, a hyper-frequency connectivity structure of a choir singing a canon with different voices is displayed (adapted from Müller et al., 2018a). A hyper-brain network with intra- and inter-brain connectivity emerging during an interaction of two people is schematically represented on the top left. The structure represents two hyper-brain modules or communities (coded in blue and red) comprising nodes from two different brains, as described in Müller. On the right, different interaction situations described in the Research Topic are presented (adapted from Klein et al. and Lange et al.; cf. also Müller et al. for the soccer game; the guitarist duo on the top right, Copyright © Arne Sattler).

## Neural synchrony and network dynamics in social interaction and communication behavior

A recently emerging view in social and cognitive neuroscience with regard to hyperscanning methods holds that interpersonal action coordination or communicative behavior require inter-brain synchronization and specific hyper-brain network activity (cf. Müller et al., 2021). This collection begins with a Hypothesis and Theory article by Müller proposing a *hyper-brain cell assembly* hypothesis, which states that cell assemblies can emerge not only within, but also between the interacting brains, following roughly the same rules as within brains. More precisely, the *hyper-brain cell assembly* encompasses and integrates oscillatory activity within and between brains, and represents a common hyper-brain unit that has a certain relation to social behavior and interaction. The suggested hyper-brain cell assembly assumes simultaneous firing of neural cells in two or more brains supported by intra- and inter-brain synchronization patterns and their continual adjustment to each other. Hyper-brain modules or communities, comprising nodes across several brains, are considered as possible representations of the hypothesized hyper-brain cell assemblies that can also have a multidimensional or multilayer structure and operate at different frequencies in their complex interplay. Müller concludes that the neuronal dynamics during interpersonal interaction and communication behavior is brain-wide, i.e., it is based on common neuronal activity of several brains or, more generally, of the coupled physiological and sensorimotor systems including brains.

Social interaction in general, and communication in particular, are dynamic processes with constant updating and adaptation of interaction/communication information and behavioral strategies, where the interacting agents are dynamically coupled rather than simply aligned (Hasson and Frith, 2016; Tognoli et al., 2020; Dumas and Fairhurst, 2021; Müller et al., 2021). As shown in a number of hyperscanning studies, such dynamic interaction is supported by brain-to-brain coupling and network dynamics (Müller et al., 2018b; Müller and Lindenberger, 2019, 2022). In a fNIRS hyperscanning study, Wang et al. used sliding window approach and k-mean clustering to investigate the temporal occurrence of the inter-brain states and network dynamics during two different group communication tasks (creative vs. non-creative). The authors found that states that occurred less frequently than others had higher network global efficiency and a shorter characteristic path length. These were termed efficient interbrain states, compared to inefficient ones, which in turn occurred very frequently. At the same time, the occurrence of efficient interbrain states and state transitions during creative communication was significantly more frequent than during non-creative communication, indicating a more active and integrated neural network during a creative task. Moreover, efficient interbrain states correlated positively with collaborative behavior and group performance. The authors conclude that there is a close correspondence between inter-brain network states and cooperative social behavior, both of which are more flexible during creative than non-creative communication.

Theory of Mind (ToM) is a construct in cognitive and developmental psychology introduced by Premack and Woodruff (1978) that indicates the ability of an individual to represent other people's mental states and drives the capacity for social interaction. ToM is not a singular skill and relies on multiple subprocesses, including, but not limited to, distinguishing self from other. Mossad et al. used fMRI (functional magnetic resonance imaging) and MEG (magnetoencephalography) neuroimaging techniques to explore the neural mechanisms of ToM abilities. In the study, where the participants had to describe videos containing three moving shapes designed to depict either social interactions or random motion (control condition), they observed increased fMRI activation in frontal-parietal regions in the social compared to the control condition, and the recruitment of ToM networks in the social condition in theta, beta, and gamma bands of MEG signals. More precisely, the right supramarginal, and angular gyri (right temporal parietal junction), right inferior parietal lobe, and right temporal pole were recruited in the first 5 s of the MEG experiment. Brain regions such as the superior frontal gyrus and the bilateral amygdalae were recruited in the second time window (5–10 s). While the earlier processes or networks were detectable in all three frequency bands, the later ones only occurred during the oscillatory activity in the beta band. Combining the strengths of the spatial resolution of fMRI and temporal resolution of MEG allowed the authors to delineate the mechanism by which ToM processing unfolds over time in a frequency-specific manner.

The ability to perceive, notice and pay attention to one's internal body state, including visceral feelings, has been defined as Interoceptive Attentiveness (IA), which represents one of the dimensions of interoception (Schulz, 2016). In a functional near-infrared spectroscopy (fNIRS) hyperscanning study, Angioletti and Balconi explored the effect of explicit IA manipulation on hemodynamic brain responses during a motor synchronization task involving interpersonal coordination framed with a social goal. An increased oxygenated hemoglobin fNIRS response in the prefrontal cortex (PFC) was found when inducing an explicit focus (IA) on the breath during the socially framed motor task requiring synchronization. Overall, the authors showed that hemodynamic activity is significantly enhanced in brain regions that support sustained attention, reorientation of attention, and social responsiveness when a joint task is performed and participants focus on their physiological body reactions.

Group cohesion can also be influenced by emotions shared in a group of people, indicating that cohesion is a multi-faceted process comprising different components or relations (Casey-Campbell and Martens, 2009). Chabin et al. investigated interbrain coupling in a group of people attending a concert and focus on the emotional dynamics of the group as a whole. The authors identified specific moments in the concert that evoked strong or weak emotions, as well as strong or weak group emotional cohesion. They found that synchrony between listeners' brains in the theta frequency band is mainly associated with the experience of high music pleasure and that emotional cohesion in the group can enhance interbrain synchrony. However, the emotional cohesion of the group is not solely responsible for inter-brain synchrony in this context. Sharing a high level of pleasure related to music presumably elicits similar brain activity in several group members, thus enhancing interbrain synchrony in the group.

## Sensorimotor synchronization and intrapersonal coordination in music ensembles and other groups

Team sport implies teamwork with a precise interpersonal coordination in a common timeframe. Such a teamwork or social group interaction can best be described in terms of dynamical system theory or generalized synchronization (Rulkov et al., 1995). On the one hand, dynamical systems are unpredictable, since their trajectories are extremely sensitive to their initial states. On the other hand, they may synchronize to a common chaotic trajectory if they are coupled to each other (Pikovsky et al., 2003; Kinzel et al., 2010). The generalized synchronization concept assumes that the behavior of several interacting individuals or subsystems can be strikingly different, but each one acts in function of the others. Müller et al. used this concept to investigate the influence of rhythmic auditory stimulation (RAS) on soccer performance. The authors provide quantitative evidence that the connectivity between teammates, expressed by fast and precise pass sequences with a minimal number of ball contacts for each player, and the scoring rate of male soccer teams improve significantly when playing under the influence of collective RAS. They conclude that results can be explained in terms of the dynamical system theory, non-linear resonances, and dynamic attention theory.

Synchronization in a team or group was also explored in a multi-person adaptive metronome study by Fink et al. using a specific assistive device (adaptive metronome) in five different experiments. The authors found that in all experiments, tapper synchronization with the metronome was significantly enhanced with 25–50% metronome adaptivity (percent correction based on the immediately preceding tap-metronome asynchrony), compared to no adaptation (Fairhurst et al., 2013). Furthermore, synchronization with the metronome reached 70–100% adaptivity in group experiments with auditory feedback. It was also shown that individuals who tapped less variably than the group felt more in the groove, a unified rhythmic effect or feeling that compels one to move and that is generally regarded as pleasurable (Janata et al., 2012). Moreover, subjective ratings of being in the groove, in synchrony with the metronome, in synchrony with others, liking the task, and task difficulty loaded onto one latent factor, which was termed enjoyment. Prediction of enjoyment required an interaction between auditory feedback and metronome adaptivity, with increased enjoyment at optimal levels of adaptivity (with auditory feedback only) and a marked decrease in enjoyment at higher levels of adaptivity, especially without feedback. The authors conclude that the adaptive metronome system holds promise for helping groups of people to achieve better motor and psychological alignment or synchrony in a variety of contexts.

There is neurophysiological evidence that constraints operating at both individual and joint scales have reciprocal effects: intrapersonal constraints affect processes of both intrapersonal and interpersonal coordination, and likewise interpersonal constraints (Ramenzoni et al., 2011; Miyata et al., 2017). This is in line with the joint forward model for interpersonal action coordination (cf. Figure 1), functional system theory, as well as the notion of circular causation of self-organized systems (Müller et al., 2021). Laroche et al. perturbed interpersonal sensorimotor communication in violin players of an orchestra and examined how this affected the musicians' intrapersonal movement coordination by using the motion capture of head and bow kinematics. The authors found that altering the usual interpersonal coupling scheme increased intrapersonal coordination and that the perturbation induced smaller yet more complex head movements. Moreover, the perturbation differentially increased intrapersonal coordination across different timescales. In general, the present study illustrates the sensitivity of intrapersonal body coordination to interpersonal coupling constraints in the complex and ecological context of a music ensemble.

In line with the aforementioned joint forward model (see Figure 1 for details), besides intra- and inter-personal sensory-motor coordination, action prediction for self and other play an essential role in social interaction, in general, and in the ensemble performance of music, in particular (Keller et al., 2014; Müller et al., 2021). Klein et al. asked professional violinists to play along with recordings of two folk pieces and investigated the information flow in the sounds. They used Granger causality to measure information flow and cross-correlation to measure synchronization between their performances and the recording sounds. The authors found that information flow from the recording to the musicians was much greater than vice versa, indicating that musicians can learn to predict how another musician will play next on the basis of the sounds they have just produced. In addition, they found that this information flow decreased as the violinists became more familiar with the recordings over trials. This was also accompanied by increased synchronization between the violinists over trials. The authors conclude that investigating information flow between the sound outcomes of live performing musicians could be a useful tool in more diverse and ecologically valid performance contexts.

Musical ensemble performances provide an ideal environment and a perfect model to study and gain insights into complex human group interactions (D'Ausilio et al., 2015). Synchronization patterns and emerging network structures can reflect specific roles of individual performers and a higher level of organization of all performers as a superordinate system or superorganism with a robust interplay between network topology and function (Bashan et al., 2012; Müller et al., 2021). Lange et al. investigated group dynamics of choral singing with and without physical contact (i.e., touching each other's shoulder or waist), using hyperscanning of respiratory and cardiac responses from eight professional singers. The idea of singing with touch was motivated by historical depictions of ensembles originating from the 14th to 17th centuries (cf. Figure 1). In line with previous studies (Müller and Lindenberger, 2011; Müller et al., 2018a, 2019), the authors found a significant increase in synchronization of respiratory and cardiac outcomes during singing as compared to rest (baseline). Most importantly, this synchronization in respiration across singers was stronger among different frequencies when singing with touch, with the effect of touch being stronger when all singers were singing in comparison to the partial ensemble. The findings suggest a higher level of organization of singers in the choir functioning as a superordinate system or superorganism when singers share the same goal.

Tomashin et al. used multidimensional recurrence quantification analysis on cardiac Interbeat Intervals (IBIs) to assess dyadic and group-level interactions during a drumming and a decision-making task, and compared these with the resting state baseline. The authors found that IBIs synchrony between group members was significantly higher than during baseline and also significantly higher in actual than in pseudo-groups (false-pair surrogates). Interestingly, synchrony during baseline was not significantly higher than in false-pair surrogates. Most importantly, the change in IBI synchrony from baseline to group interaction predicted a psychological sense of group cohesion measured by using cohesion questionnaire. This result was evident at both the individual and the group levels and was independent of the interaction task. However, it should be noted that the positive cohesion effect was found only for change from baseline to group interaction, whereas no significant results for cohesion were found for the groups' synchrony during the task and the effect of baseline synchrony on cohesion was significantly negative. Thus, cohesion is considered as an emergent or dynamic state that is socially modulated by group experiences or relationships and represents a multilayered construct with intertwined coupling dynamics (Marks et al., 2001; Konvalinka et al., 2011).

As mentioned above, there is neurophysiological evidence that positive emotions or pleasure may facilitate neural or physiological synchrony within a group of people (Konvalinka et al., 2011; Chabin et al., 2022). Smykovskyi et al. investigated group interaction in triads of people engaged in a movement improvisation task and explored the effect of emotional feedback on behavioral, psychological, and physiological levels. The participants were instructed to create complex, varied, and interesting movements with their right hand to express themselves. The authors showed that positive and negative emotions differently alter spontaneous human synchronous behavior (movement synchrony). On the psychological level, a significant effect of emotion was obtained on pleasure but not on arousal scores. On the physiological level, no significant effects of emotions on the cardiac activity of the triad were found. This result partially contradicts the previous findings (e.g., Konvalinka et al., 2011) and therefore, further research is required for clarification.

## Psychotherapy, music therapy, and neurodynamics of healthcare teams

As mentioned above, synchronization within and between brains appears to be crucial for interpersonal action coordination and is an important element of neural communication systems during an interaction (Müller et al., 2021; Shamay-Tsoory, 2021). Such synchronization is important not only in daily life, but also in therapy, as it gives the patient and the therapist access to each other's inner states and facilitates mutual understanding and emotional exchange (Koole and Tschacher, 2016). In their review paper, Sened et al. propose that therapy improves patients' ability to achieve such synchrony through inter-brain plasticity, i.e., recurrent activation of specific brain regions in the patient and therapist in close succession (compare the “hyper-brain cell assembly hypothesis” suggested by Müller). This can lead to a long-term improvement in the ability to synchronize and to generalize to other interpersonal relationships and other situations, ultimately leading to a reduction in symptoms. This review suggests that the inter-brain plasticity model offers a novel biological framework for understanding relational change in psychotherapy, and the enhanced capacity for synchronization and generalization underlies some of the beneficial effects of psychotherapy.

Music therapy is tied to the process of creating and experiencing music together through improvisation, listening, and reflection (Fachner, 2014). Despite the diversity of techniques and models in music therapy, they all have one thing in common in that music and the relationships developed within it are the foundation for change (Millard and Carr, 2021). Yap et al. investigated non-verbal synchrony or coordination of body movement between patient and therapist using Motion Energy Analysis (MEA) from a video source and subsequent calculation of cross-correlation between the MEA time series. This analysis revealed an increase in motion synchrony and patient leading after the music intervention, possibly due to greater familiarity between therapist and patient, as they had already spent some time together in a music therapy session, as well as improved self-regulation, thus empowering the patient. This presents a novel method for investigating non-verbal synchrony in music therapy in neuro-rehabilitation.

Healthcare team members jointly regulate their activities and operate at the collective behavioral level while coordinating their actions and interacting dynamically, interdependently, and adaptively toward common goals. Stevens and Galloway investigated the differential neurodynamics of seven two-person healthcare teams across time and brain regions during autonomous (taskwork) and collaborative (teamwork) segments of simulation training. The authors used a neurodynamic information (NI) EEG measure, describing the pauses and hesitations associated with individual uncertainty, and interbrain coherence (IBC), which is an inevitable component of social interactions. No correlation was found between NI and IBC measures, and second-by-second dynamic comparison indicated mutual exclusivity. The authors observe that the sustained expression of NI and IBC did not occur simultaneously, suggesting that team members may find it difficult to maintain synchrony between brains while reducing their individual uncertainties.

With noteworthy diversity of topics and research questions explored, the studies in this Research Topic emphasize the important mechanisms and functions of interpersonal action coordination and social interaction. They confirm that the neuronal dynamics during interpersonal interaction is brain- or system-wide, i.e., it is based on common neuronal activity synchronized across brains or, more generally, on coupled physiological and sensorimotor systems including brains. These results highlight future avenues for applications in basic research and therapeutic areas, and specify the role of hyperscanning research in the growing field of social neuroscience.

## Author contributions

All authors contributed to the writing of this editorial and approved the submitted version.

## Conflict of interest

The authors declare that the research was conducted in the absence of any commercial or financial relationships that could be construed as a potential conflict of interest.

## Publisher's note

All claims expressed in this article are solely those of the authors and do not necessarily represent those of their affiliated organizations, or those of the publisher, the editors and the reviewers. Any product that may be evaluated in this article, or claim that may be made by its manufacturer, is not guaranteed or endorsed by the publisher.
